# Accessible, All-Polymer Metasurfaces: Low Effort, High Quality Factor

**DOI:** 10.1021/acsnano.5c15415

**Published:** 2026-02-18

**Authors:** Michael Hirler, Alexander A. Antonov, Enrico Baù, Andreas Aigner, Connor Heimig, Haiyang Hu, Andreas Tittl

**Affiliations:** Chair in Hybrid Nanosystems, Nanoinstitute Munich, Faculty of Physics, 9183 Ludwig-Maximilians-University, 80539 Munich, Germany

**Keywords:** polymers, metasurfaces, bound states in the continuum, nanofabrication, low-index photonics

## Abstract

Optical metasurfaces supporting resonances with high quality factors offer an outstanding platform for applications such as nonlinear optics, light guiding, lasing, sensing, light-matter coupling, and quantum optics. However, their experimental realization typically demands elaborate multistep procedures such as metal or dielectric deposition, lift-off, and reactive ion etching. As a consequence, accessibility, large-scale production, and sustainability are constrained by reliance on cost-, time-, and labor-intensive facilities. We overcome this fabrication hurdle by repurposing poly­(methyl methacrylate), which is usually employed as a temporary resist, as the resonator material, thereby eliminating all steps except for spin-coating, exposure, and development. Because the low refractive index of the polymer limits effective mode formation, we present a bilayer recipe that enables the convenient fabrication of a freestanding membrane to maximize the index contrast with its surroundings. Since etching induced defects are circumvented, the membrane features high quality nanopatterns. We further examine the suspended membrane with scanning electron microscopy and extract its position-dependent spring constant and pretension with nanoindentation experiments applied with the tip of an atomic force microscope. Our all-polymer metasurface hosting bound states in the continuum experimentally delivers high quality factors (up to 523) at visible and near-infrared wavelengths, despite the low refractive index of the polymer, and enables straightforward geometry-based tuning of both line width and resonance position. We envision this methodology to facilitate accessible, high performance metasurfaces with specialized use cases such as material blending, angled writing, and mechanically based resonance tuning.

## Graphical abstract

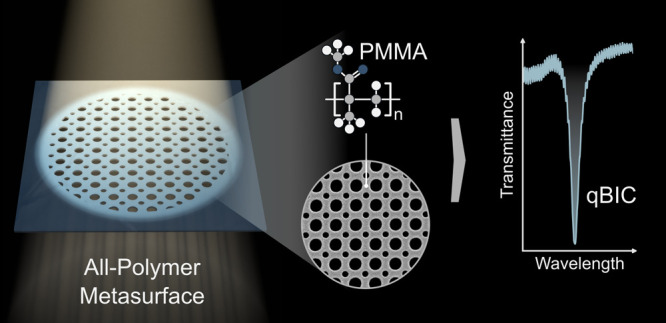

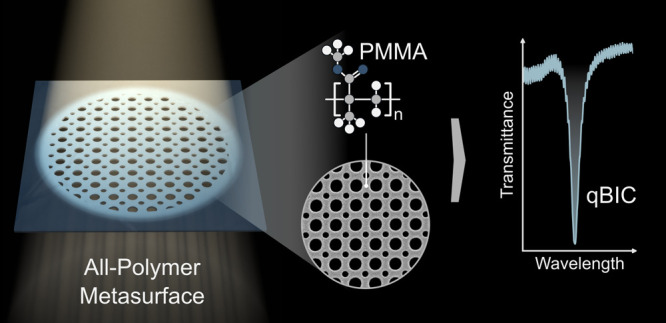

Optical metasurfaces are ultrathin flat photonic structures that owe decisive parts of their optical properties to their artificial subwavelength features, enabling efficient manipulation of light at the nanoscale beyond the capabilities of conventional bulk materials.^1–3^ Inspired by findings such as the generalization of Snell’s law by Yin *et al.*,^4^ metasurfaces have reshaped the landscape of flat optics, achieving landmarks in the level of control over phase,^4–7^ polarization,^6,8^ amplitude^5,9,10^ and dispersion^11^ of incident waves with subwavelength resolution. In particular, they can be engineered to strongly suppress scattering losses and support resonances with high quality (*Q*) factors through various physical mechanism.^12–14^ One of these mechanisms has recently attracted increasing attention: metasurfaces supporting bound states in the continuum (BICs) offer a high degree of control over radiative losses.^15–17^ A true BIC is a perfectly confined mode without any radiation yet residing in the continuum of radiating modes. By perturbing this ideal lossless mode, a resonance known as quasi-BIC (qBIC) becomes accessible in the far field. QBICs have enabled a wide range of applications including nonlinear optics,^18^ light guiding,^19^ lasing,^20^ sensing,^21–24^ light-matter coupling^25–28^ and quantum optics.^29^

Although qBICs have been demonstrated in both metallic^30–32^ and dielectric^21,33,34^ structures, the significant resistive losses in plasmonic resonators have led to the widespread use of high-index and low-loss dielectrics such as silicon and germanium.^35,36^ However, restricted material diversity limits the scope of applications, missing out on the potential other materials might offer: Not only are low-loss, high-index materials rare at visible wavelengths,^37,38^ but patterning them into high-quality nanostructures demands a sophisticated infrastructure that is cost-, time- and labor-intensive, ultimately limiting large-scale commercialization.^39^ Moreover, etching-based procedures are prone to fabrication errors such as geometric perturbations^40–42^ and material redeposition.^43,44^ At the same time, the complex fabrication demands substantial energy and material consumption, along with the intensive use of gases and chemicals, resulting in a significant ecological footprint and safety risk. Gases like chlorine and chemicals like hydrofluoric acid, for instance, require careful disposal and pose the risk of leaks or hazardous accidents.^45,46^

Polymers are safe, affordable and easy to process.^47^ In fact, they already play an integral role in state-of-the-art lithography processes.^48,49^ In the form of resists, they are used to transfer patterns onto the target material. Typically, they serve a temporary purpose and are removed from the final device during the lift-off process. A conventional fabrication workflow of metasurfaces is schematically illustrated in Figure 1b: the deposition of a metal or dielectric is followed by spin-coating of the resist, exposure with photons or electrons, resist development, deposition of the etching mask, lift-off, etching, and the removal of the mask. Among common polymers, poly­(methyl methacrylate) (PMMA) is arguably the most available resist. It is well-established, extensively studied and has been effectively combined with other resists for multilayer precedures.^50,51^ However, the low refractive index (RI) of polymers hampers their utility as integral constituents of resonant metasurfaces since the dissipation of optical power into the substrate inhibits the effective formation of confined modes. Metallic substrates such as gold or silver have been proposed to prevent mode leakage by interaction with surface plasmons.^52,53^ For instance, Kulkarni *et al.* demonstrated qBICs with *Q* factors up to 305 at visible wavelengths emerging from a polymer resist film placed on a silver substrate.^54^ But the ohmic losses in the substrate eventually limit the potential of BICs, restricting the diverging nature of their *Q* factors.^53^ In addition, the use of metallic mirrors restricts the device to operate in reflection, whereas many applications including cascaded metasurfaces and device-integration^55,56^ favor transmission. A suspended polymeric photonic crystal nanocavity presented by Martiradonna *et al.* circumvented substrate induced losses as well as restriction to reflection mode altogether, but came at the cost of reversion to elaborate fabrication procedures involving hazardous chemicals.^57^ To date, a simple and cost-effective fabrication technique for a suspended nanopatterned polymer membrane is still pending.

**Figure 1:**
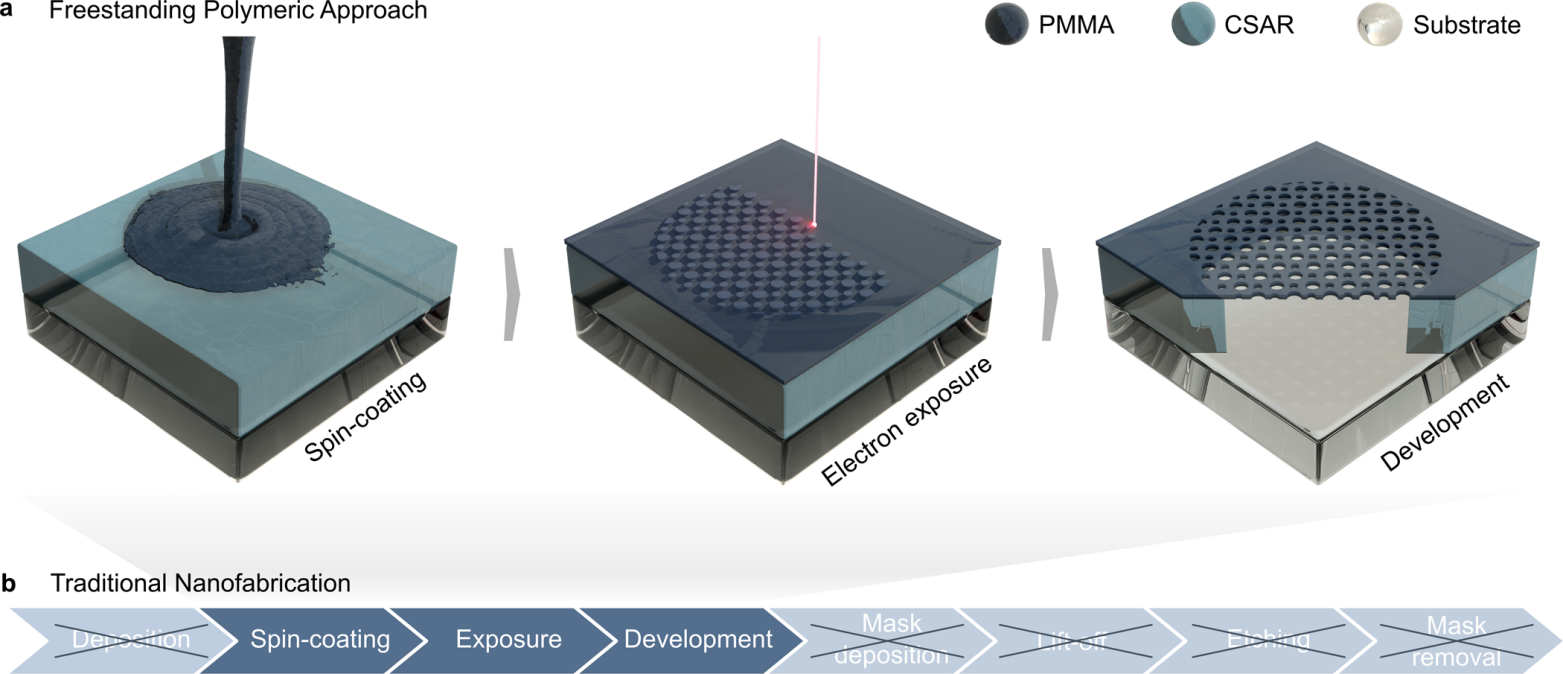
Simplified fabrication workflow. (a) Schematic illustration of the nanofabrication workflow for an all-polymeric metasurface, solely consisting of spin-coating, exposure, and development. This greatly simplifies the traditional fabrication process as shown in (b).

In this work, we realize an all-polymer metasurface based on the qBICs. Our approach drastically simplifies the nanofabrication process, solely relying on spin-coating, exposure and development (Figure 1a). The result is a freestanding PMMA membrane whose photonic behavior is independent of parasitic diffraction and losses associated with the substrate. We numerically simulate a C_4_-symmetric perforated membrane hosting both, an in-plane electric and a magnetic BIC. Then, we outline our bilayer fabrication procedure before we verify the formation of high quality nanopatterns in suspended polymer films with scanning electron microscopy (SEM). Nanoindentation experiments applied by the tip of an atomic force microscope (AFM) further retrieve the membrane’s position-dependent spring constant and pretension. Finally, we experimentally demonstrate that the patterned polymer films support qBICs at visible and near-infrared wavelengths with *Q* factors as high as 523 along with geometry driven tunability of the resonance’s position and line width.

## Results and Discussion

### Numerical Modeling of a Freestanding Metasurface with BZF-BIC

As a basis for our platform, we utilized a design consisting of periodic hole arrays in a freestanding PMMA membrane, where the quadratic unit cell encloses a void at its center (Figure 2a). Assuming the absence of internal losses and for normal incidence, the bound state is nonradiative with an infinitely high *Q* factor as long as the holes are identical in size. Such a perfectly confined state cannot couple to a free space light. In order to access the otherwise dark state, we performed Brillouin zone folding (BZF) in momentum space by periodically varying the radii of adjacent holes. The perturbation parameter $\alpha =\frac{r_{1}-r_{2}}{r_{1}}=\frac{\Delta r}{r_{1}}$ quantifies this variation. This new configuration extends the unit cell by a factor of √2 and introduces a radiative leakage channel, converting the true BIC into a qBIC with high but finite *Q* factor. In the far field transmittance spectrum (Figure 2b), this manifests in the emergence of two BZF induced qBICs; we distinguish them by their in-plane field distribution as an electric and magnetic mode. Their line width and resonance position depend on α, which exemplarily demonstrates the large degree of control BIC based structures allow to exert over the radiative loss channel.^58^ The resonance wavelength can be controlled by other geometrical parameters^21,25,28,38^ —here, the resonance is designed to reside at visible wavelengths, with a membrane thickness *t* of 300 nm and unit cell periodicities *p* of 410 nm (unperturbed design) and 580 nm (perturbed design). The holes are chosen such that the combined area *A* of two holes is conserved upon changes in α, *i.e. A* = 2π*r*_0_^2^ where *r*_0_= 135.83 nm for the unperturbed and *A* = π (*r*_1_^2^ + *r*_2_^2^) for the perturbed configuration. It directly follows that *r*_2_ = *r*_1_ (1 – α) and $r_{1}=\sqrt{A{\left[\pi (1+{\left(1‐\alpha \right)}^{2}\right]}^{-1}}$ . The RI of PMMA was obtained from in-house ellipsometry measurements (Supporting Information 1) and can be approximated as 1.5. Varying the membrane thickness *t* is an effective means to tune the *Q* factors of both modes (Supporting Information 2). Since the magnetic qBIC features very narrow line widths that are difficult to access experimentally, we focus on the electric mode throughout this work. Figure 2c indicates the electric near field enhancement of the perturbed configuration for α = 0.5. The fields are efficiently concentrated in the voids (maximum enhancement of *|E*/*E*_0_ *|* ≈ 18, where *E*_0_ denotes the amplitude of the incident electric field), which makes the platform a promising candidate for effective near field light-matter coupling.^28,59,60^ Considering momentum space, we demonstrate that at the *Γ*-point (*i.e. k*_*x*_ = *k*_*y*_ = 0), there are two degenerate electric modes, which enables their coupling to the far field. In contrast, nondegenerate modes in a structure with C_4_ rotational symmetry cannot radiate.^61^ Deviations from the *Γ*-point break C_4_ rotational symmetry and lift the qBIC’s degeneracy, resulting in two electric modes depending on the polarization. We labeled them mode 1 and mode 2. Interestingly, mode 1 exhibits a pronounced angle-stability in the directions where *k*_*x*_ = 0 or *k*_*y*_ = 0, respectively (Figure 2d), while the *Q* factor remains roughly constant (Figure 2e). Mode 2, in contrast, is significantly more sensitive along those directions. This implies that depending on the polarization, the system can be chosen to exhibit either robustness or sensitivity upon changes in the angle of the incident light. Further simulations of the far field polarization (Figure 2f) unveil the topological nature of the modes. Both vortices show integer winding numbers of the polarization vectors around the vortex centers, corresponding to topological charges of −1, which points at the underlying physics of qBICs.^62^ Analogous considerations for the magnetic qBIC can be found in Supporting Information 3.

**Figure 2:**
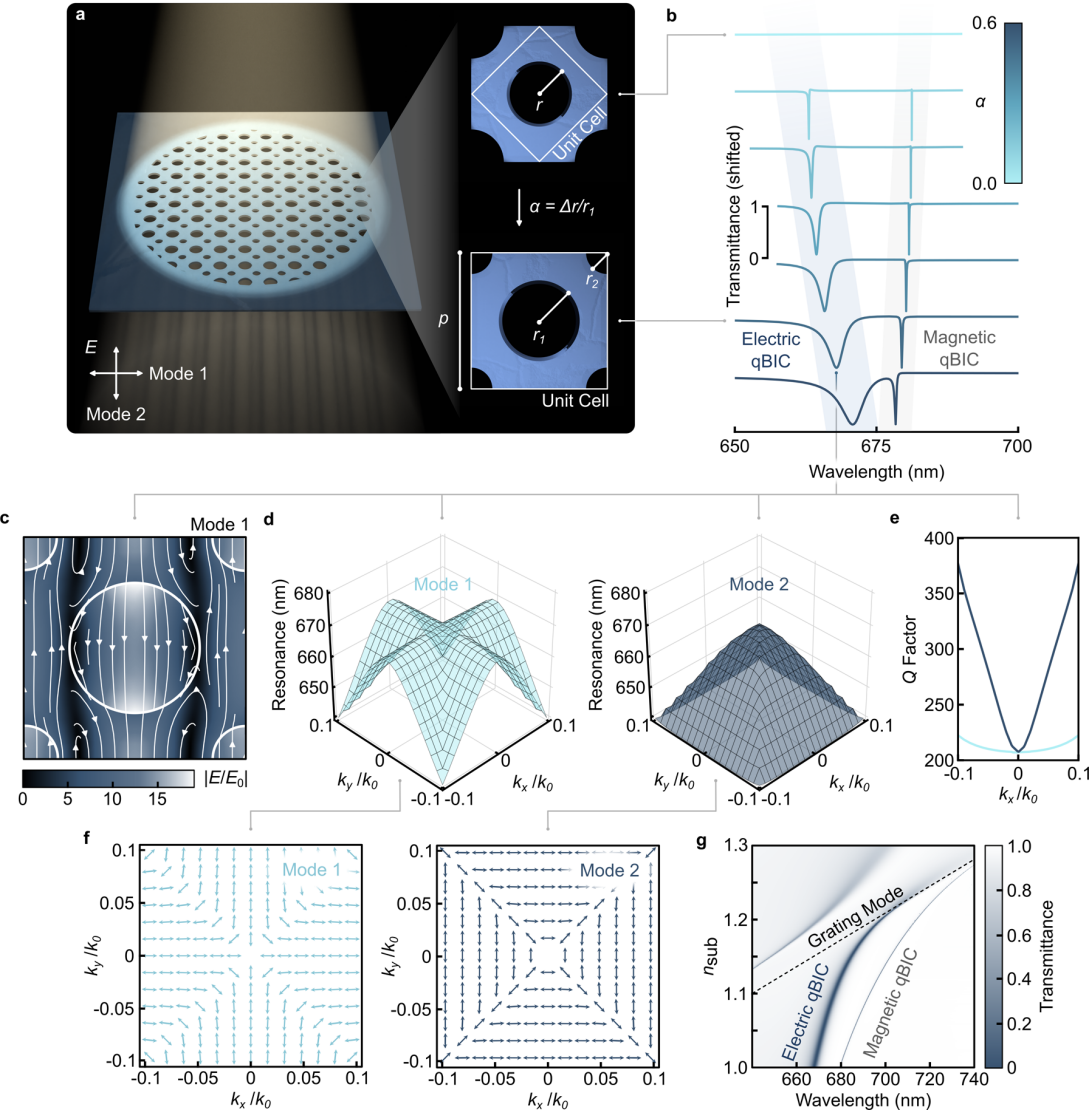
Numerical modeling of a freestanding metasurface with BZF-BIC. (a) Artistic rendering of a freestanding membrane featuring hole arrays that support true BIC (top) and BZF-quasi (bottom) BIC. (b) Transmittance spectra for different perturbations α. (c) Electric near field enhancement *|E*/*E*_0_ *|*, (d) resonance position, and (e) *Q* factor in momentum space as well as (f) far field polarization for the two electric modes that are degenerate at the *Γ*-point. (g) Transmittance spectra depending on the RI of the substrate *n*_*sub*_.

Our simulations confirm that the BIC mechanism enables effective mode confinement in freestanding PMMA membranes with *Q* factors competitive with higher-index materials despite the polymer’s low RI of roughly 1.5 (Supporting Information 4). However, most commonly, metasurfaces are fabricated on substrates. Not only does this grant mechanical stability but also simplify the fabrication procedure.

This is where the use of low-index polymers poses a significant hurdle: analogous to conventional optical gratings, periodic metasurfaces give rise to discrete diffraction orders. Such grating modes, also known as Rayleigh-Wood anomalies,^63,64^ are a consequence of the structure’s periodicity *p* and their wavelength λ_*m*_ is given by $\lambda_{m}=\frac{n_{\mathrm{sub}}p}{m}$ for normal incidence, where *n*_*sub*_ is the refractive index of the substrate and *m* is an integer indicating the diffraction order. The qBIC should reside at wavelengths longer than the diffraction cutoff of the first diffraction order to prevent radiative leakage into additional non-zero diffraction channels. Given the contrast in RI of PMMA with commonly used substrates, such as fused silica with *n*_*sub*_ ≈ 1.45, is rather small, the resonance and diffraction edge occur in close spectral proximity. For a patterned PMMA membrane resting on a substrate, even low values of *n*_*sub*_ result in strong damping of the qBIC (Figure 2g). Due to the low RI contrast and thus the dissipation of optical power into the substrate, this cannot be prevented by geometrical corrections. Consequently, the challenge is to either employ ultralow-index substrates^65,66^ or, preferably, to manufacture a freestanding polymer membrane. While there have been demonstrations of freestanding membranes, for instance, consisting of silicon,^28^ silicon nitride,^59,60^ or alumina,^67^ the potential of polymers, which are easier to process, has not yet been fully harvested.

### Experimental Realization and Examination of Freestanding PMMA Metasurface

In order to build a freestanding membrane without reverting to cumbersome deposition and etching steps, we propose a top-down bilayer resist recipe: We spin-coated a 300 nm thick PMMA film on top of a *ca.* 1250 nm thick sacrificial layer consisting of CSAR resist. The PMMA was solved in ethyl lactate to prevent mixing with the underlying layer. The thickness of the PMMA membrane is determined by the spin-curve (Supporting Information 2) and the mechanical integrity of the material. Freestanding membranes as thin as 200 nm could be achieved. During subsequent electron beam exposure, the different sensitivities of the two polymers were exploited. While applied doses ranging from 200 to 250 μC/cm^2^ patterned the nanohole array into the PMMA layer, the underlying CSAR was overexposed instead. This selective exposure allowed the sacrificial CSAR layer to be entirely removed through the porous PMMA film during development, releasing a freestanding membrane (Figure 1a). More details can be found in the methods section and in Supporting Information 5. Owing to the use of polymers, neither deposition, etching, nor lift-off were required. Reducing the required number of steps also reduces the procedure’s proneness to errors.

We examine the patterned and suspended membrane in two ways: first, Figure 3a shows SEM images of the final device. The metasurface’s circumference was designed to be circular rather than rectangular to homogenize the strain profile, as sharp corners were found to promote fractures in the polymer film (Supporting Information 6). The diameter measures 30 μm, whereas the largest fabricated metasurface reached 40 μm. The film in Figure 3b additionally features a notch to verify the removal of the sacrificial layer and the suspension of the metasurface. Noteworthy, the hanging PMMA shows little to no bending despite its thickness of only 300 μm, while the holes exhibit high quality with little imperfections. Since the polymeric approach circumvents any pattern transfer, the PMMA was not damaged by etching. Consequently, the precision of the final structure almost solely relies on a high-resolution lithography step. The distance between the metasurface and the substrate is sufficiently large to prevent any near field interaction, ensuring that the photonic response is not influenced by the presence of the substrate. Second, to study the mechanical properties of our all-polymer metasurface, AFM measurements were performed, both in tapping and contact mode, in order to extract the spring constants and pretensions of the freestanding membrane, respectively. Unlike in Figure 3a, the image taken in tapping mode (Figure 3c) shows a depression at the location of the metasurface relative to its surroundings, suggesting that the mechanical load applied by the tip bends the elastic membrane downward. Furthermore, we conducted contact mode retrace curves at several positions across the metasurface (dots in Figure 3c) with an initial force set to around 0.6 μN. Starting from the border and moving toward the center, each position was measured ten times, resulting in the averaged force–displacement (*F*–δ) curves exemplarily presented in the inset of Figure 3e (all curves are shown in Supporting Information 4). The measurements suggest that the membrane stiffness decreases with the distance *x* from the bulk material around the metasurface, indicated by the shallower slopes. To obtain the elastic membrane deformation δ, we subtract the cantilever deflection *Δz*_*c*_ from the displacement of the scanning piezotube of the AFM instrument Δ*z*_*piezo*_ as δ = Δ*z*_*piezo*_ – *Δz*_*c*_ (Figure 3d). *Δz*_*c*_ can be obtained by recording a reference retrace curve on silicon (Supporting Information 7). To describe the bending of our metasurface, we employ a simplified model of Kirchhoff plate theory that relates the applied force to the deformation of the membrane.^68^ Since the metasurface radius *R* of 15 μm is much larger than its thickness *t* of 300 nm, we consider the relation between loading force *F* and displacement δ in the limit $\frac{t}{R}\ll 1$ , given by the following form:^69,70^

1 $$F=\left[\frac{4\pi E}{3\left(1-\nu^{2}\right)}\frac{t^{3}}{R^{2}}+\pi T\right]\delta$$

where *E* is Young’s modulus, ν the Poisson ratio of PMMA and *T* is the pretension of the fabricated membrane. Since the relationship between *F* and δ is linear for this approximation, we fit a slope to the measured retrace curves, which corresponds to the membrane spring constant $\kappa =\frac{\partial F}{\partial \delta }$ . As seen in the retrace curves, the extracted spring constant is position-dependent and decreases sharply with increasing distance from the border, ranging from 120 to 20 N/m (Figure 3e). Both, the order of the spring constant’s magnitude and its qualitative behavior across the membrane are similar to previous conducted studies on suspended nanomembranes consisting of polymeric layers with gold nanoparticle intralayers.^71,72^ We observe that the contribution to the spring constant stems mainly from the pretension, *T*, which is remarkably high for such a soft material. We attribute these values to fabrication processes such as baking, which could induce stress on the PMMA before suspension.

**Figure 3:**
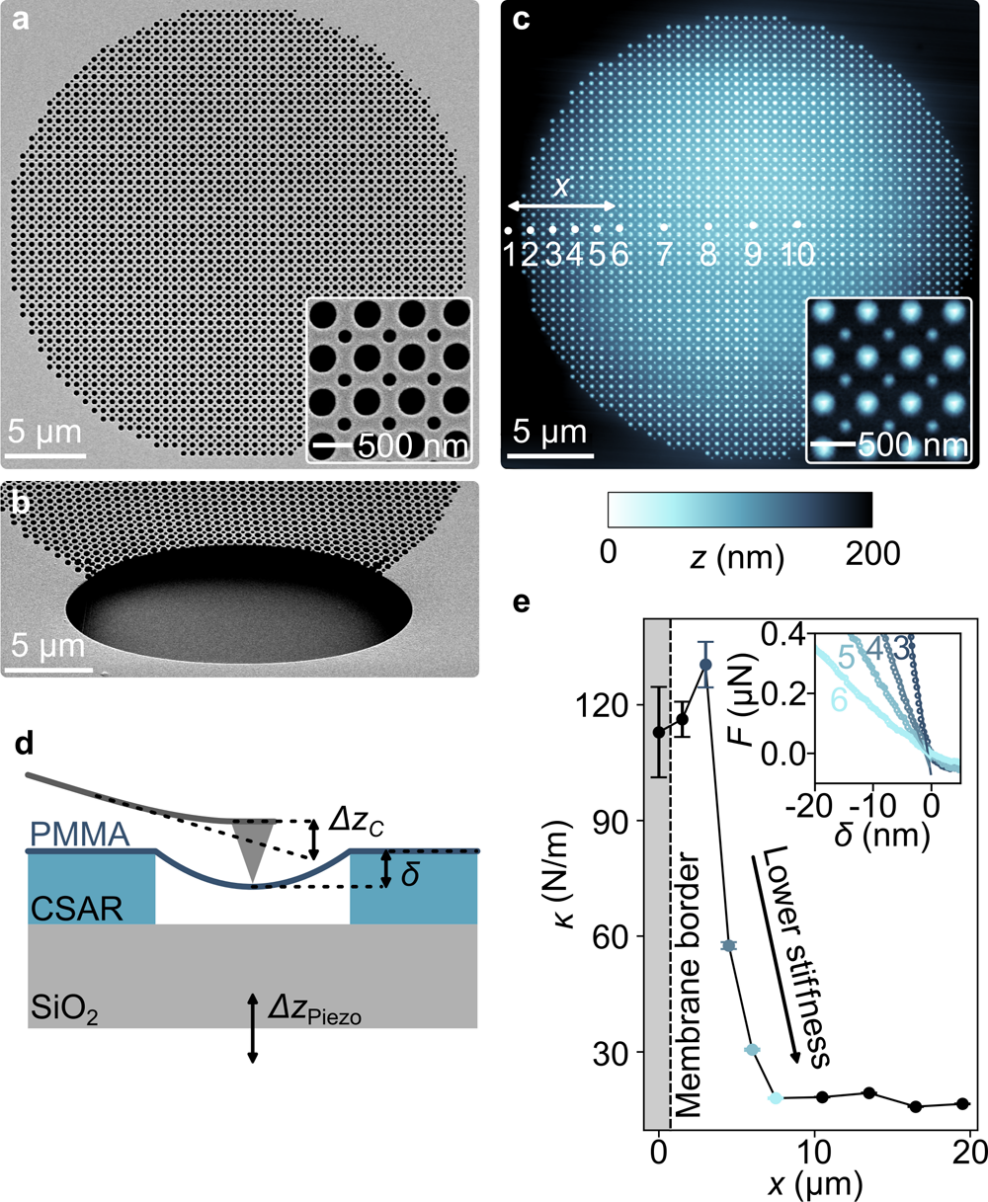
Fabricated membrane under a mechanical load. SEM images of (a) a fabricated metasurface with α = 0.5 and (b) a metasurface featuring a notch to illustrate the suspension (viewing angle of 45°). (c) Measured topography of the membrane. The white dots indicate the positions of the retrace curves shown in (e). (d) Schematic diagram of the nanoscopic bending test experiment. (e) Membrane spring constant κ­(*x*) as a function of the distance *x* from the surrounding bulk material obtained from fitting eq 1 to retrace curves. The inset exemplarily shows four of these force–displacement (*F*–δ) curves measured at different positions, as indicated in (c). The dashed black line delineates the border of the metasurface from the surrounding bulk polymer. Error bars show the standard deviation of κ fitted to each retrace curve.

### Optical Characterization of the Metasurface

Experimental spectra were recorded in transmittance. The measurements in Figure 4a show good agreement with the simulations (Figure 2b): while in the unperturbed case with identical hole radii (α = 0) the mode is completely decoupled from the radiative continuum and thus absent in the spectrum, the radius perturbations (α > 0) open radiative leakage channels, and the electric mode resonance emerges. The highest *Q* factor measured was 523 (Figure 4b) as determined by fitting a Fano resonance. We attribute deviations from the simulations to nonideal illumination conditions as well as finite size effects.^73^ Besides control over the line width, we further demonstrate tunability of the resonance position by scaling the unit cell’s lateral dimensions by a scaling factor *S*. Figure 4c shows the corresponding transmittance spectra: as *S* increased from 0.8 to 1.3, the electric qBIC responds by shifting from 551 to 838 nm; that is, from visible to near-infrared wavelengths. Note that the spectra were individually normalized such that the minimum and maximum transmittance equal 0 and 1, respectively (raw data are reported in Supporting Information 8). The geometry driven tunability of the qBIC as well as its stability against mechanical drifts and thermal heating (Supporting Information 9) enables high adaptability to application-specific demands, such as field-deployed sensors that need to be stable even at elevated temperatures.

**Figure 4:**
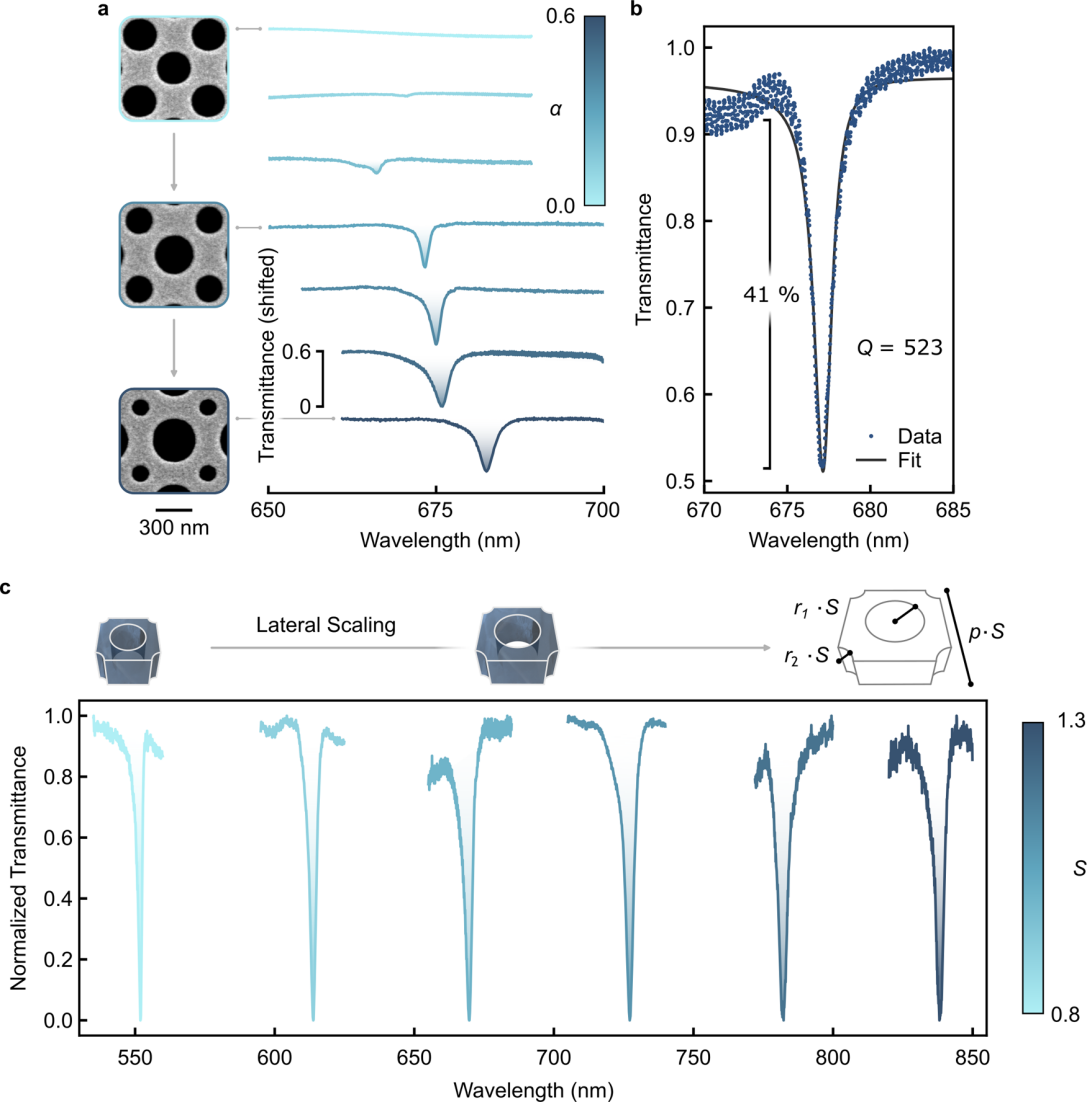
Experimental spectra. (a) SEM close ups of various perturbation parameters α along with corresponding transmittance spectra of the electric mode. (b) Resonance with highest measured *Q* factor of 523 and amplitude of more than 40%. (c) Transmittance spectra normalized from 0 to 1 from the visible to near-infrared region obtained from lateral scaling of the unit cell with α = 0.5 by a factor *S*.

Such sensors could be used for label-free refractometric sensing of surface-adsorbed biomolecules, such as proteins. In simulation, we investigate the surface sensitivity $S_{S}=\frac{\Delta \lambda }{t_{A}}$ of our membrane, where Δλ describes the resonant shift upon analyte binding and *t*_*A*_ the thickness of a thin film with RI *n*_*A*_ = 1.4. We find *S*_*S*_ = 0.96 for α = 0.5 for the electric qBIC resonance (Supporting Information 10). Previous studies demonstrated *S*_*S*_ = 1.12 for an edge qBIC in silicon pillars coated with an alumina nanofilm.^74^ Given that our platform features simple to process low-index polymers and reduced fabrication complexity, the performance of our all-polymeric approach is competitive and could find application in cost-effective thin-film nanosensors.

## Conclusion

In essence, we developed an all-polymer metasurface with geometrically tunable qBICs at visible and near-infrared wavelengths with *Q* factors up to 523. Our bilayer fabrication methodology—consisting solely of spin-coating, exposure, and development, offers advantages over conventional fabrication procedures in terms of facility requirements, time and labor costs, and environmental footprint. At the same time, since etching induced defects are circumvented, it delivers high quality nanopatterns. Utilizing photolithography instead of electron beam lithography could also significantly reduce the exposure time, paving the way for high-throughput commercialization. However, because PMMA requires radiation with wavelengths shorter than 300 nm for effective chain scission, either exposure with appropriate deep-ultraviolet light or the addition of chemical agents to extend PMMA’s chain-scission sensitivity to longer wavelengths would be required.^75^ The suitability of CSAR for photolithography remains to be investigated. Alternatively, established dual-layer photolithography procedures using different resist combinations, such as BCI 3511 and SUN-9i, could also be considered.^76^ Moreover, we envision extensions of our method to enable specialized use cases such as material blending,^77^ for instance with emitting particles for lasing applications,^54^ or with alumina nanoparticles to increase the RI^78^ for use in aqueous environments. Such particles could be blended with PMMA based on known solution-casting techniques.^78^ Angled electron exposure could further allow for chiral photonics. Our suspended membrane metasurface is further able to withstand loads of at least 0.6 μN without rupturing and exhibits spring constants of 20 N/m and higher. Such mechanical stability suggests that our platform could be directly used to build robust, yet inexpensive nanosensors resilient to environmental perturbations such as vibrations and other forms of mechanical stress.^79^ The polymer’s stability could be further increased by cross-linking. The membrane’s flexibility may further offer a promising basis for reversible mechanical tunability of the geometric parameters and thus the optical response, potentially in conjunction with simultaneous strain-engineering of materials such as two-dimensional transition metal dichalcogenides for photoluminescence enhancement.^80^ The strong near field enhancement of the membrane would simultaneously enhance the light-matter interaction with such materials.^28,59,60^ The two-dimensional material could be transferred onto the exposed polymer stack before developing the device to release the membrane. In this case, drainage channels would be required to allow the polymers beneath the flake to be removed. We hope to contribute to the development of accessible, high performance metasurfaces with a variety of applications.

## Methods

### Numerical Modeling

The numerical simulations in Figures 2b,c,g, S2a, S3a, S4a, and S10a were performed in CST Studio Suite 2023 (Daussault Systèmes), a commercial finite element solver. Operating in frequency domain, we assumed periodic boundary conditions in x- and *y*-directions and configurated adaptive mesh refinement. The RI of PMMA was imported from in-house ellipsometry data (Supporting Information 1). To solve the eigenstate problem (Figure 2d,e and S3b,c), we utilized the electromagnetic wave frequency domain module of COMSOL Multiphysics in 3D mode together with the COMSOL eigenstate solver. The tetrahedral spatial mesh for Finite Element Method was automatically generated by COMSOL’s physics-controlled preset. Simulations were performed within a rectangular spatial domain containing a single metasurface unit cell with periodic boundary conditions applied to its sides. Analogous settings were applied in the CST eigenmode solver calculations in Figure S2b. To calculate the far field polarization (Figure 2f and S3d) of the eigenstates the overlap integrals between the eigenmodes displacement current and plane waves were estimated using a previously developed approach.^81^

### Fabrication of Freestanding Polymer Membranes

Prior to the applications of the resists, the adhesion promoter SurPass 4000 (MicroResist) was coated (500 rpm for 30 s) onto cleaned fused silica substrates (MicroChemicals), washed off with isopropanol, and spin-dried (3000 rpm for 30 s). Next, two layers of CSAR 62 (AR-P 6200.13, Allresist) were successively applied (1000 rpm for 1 min) and baked at 170 °C for 5 min, adding up to a *ca.* 1250 nm thick sacrificial layer. A 300 nm thick layer of PMMA (AR-P 679–04, Allresist) was then added on top (3600 rpm for 1 min) and the mixture was baked at 150 °C for 3 min. The conductive polymer Espacer 300Z (Showa Denko K.K.) finalized the spin-coating procedure (2000 rpm, 1 min) and prepared the sample for the following electron beam lithography treatment. To this end, an eLINE Plus system (Raith) operating at 20 kV with 15 μm aperture wrote the metasurface geometry into the films with doses ranging from 200 to 250 μC/cm^2^. Finally, the patterned sample was immersed, first, in a 7:3 isopropanol-water blend for 20 s to develop the PMMA and, second, in cold Amyl Acetate (Supelco) at 6 °C for 10 s to remove the underlying CSAR. The process was eventually stopped in Novec 7100 (Sigma-Aldrich), which evaporates readily without requiring blow-drying. Alternatively, the samples can be rinsed in isopropanol and dried with a nitrogen jet. The procedure yielded a patterned, freestanding polymer membrane. Supporting Information 5 shows a schematic illustration of the procedure.

### SEM Imaging

SEM images were taken with a Gemini device from Zeiss operating at 2–3 kV. Beforehand, a few nanometer thin palladium–gold film was sputtered on the samples to increase the contrast and reduce charging effects. Afterward, some images were postprocessed by carefully adjusting the brightness and contrast.

### Optical Characterization

The spectra of the metasurfaces were recorded with a commercial white-light confocal optical microscope (WiTec alpha 300 series, Oxford Instruments) in transmittance mode. Illumination was directed from below with linearized collimated light from a broadband halogen lamp (OSL2, Thorlabs), which was collected by a 20x objective (Zeiss, NA = 0.4) and coupled to a multimode fiber. The collected light was dispersed by a diffraction grating with 600 mm^–1^ groove density (1800 in Figure 4b) and subsequently detected by a silicon CCD sensor. To remove undesired features from the sample or the beam path, we referenced all of our measurements with the transmittance spectra of the unpatterned sample. Each individual spectrum was obtained from 10 accumulations with an integration time of 0.5 s per accumulation.

### Determination of *Q* Factors

Temporal Coupled Mode Theory^82,83^ was employed to determine the *Q* factors of the resonances (as in Figure 4b). To this end, we fitted the following equation for the transmittance *T* to the spectra:

2 $$T={\left|e^{i\phi }t_{o}+\frac{\gamma_{r}}{\gamma_{r}+\gamma_{i}+i\left(\lambda -\lambda_{res}\right)}\right|}^{2}$$

where *e* ^*iϕ*^ *t*_*o*_ describes the background transmission, λ_*res*_ the resonance wavelength and the loss rate γ = γ_*r*_ + γ_*i*_ includes the radiative (γ_*r*_) and intrinsic (γ_*i*_) losses. The *Q* factor can be subsequently determined as $Q=\frac{\lambda_{res}}{2\gamma }$ .

### AFM Experiments

The AFM measurements were conducted using a commercially available AFM-based scattering scanning near field optical microscope (NeaSNOM, Attocube Systems), operating in both tapping mode for imaging (Figure 3c) and contact mode for recording retrace curves (Figures 3e and S7). The AFM tip (Arrow-NCPt, NanoWorld) used has a cantilever spring constant of κ_*c*_ = 42 N/m and a resonance frequency of Ω ≈ 250 kHz in tapping mode. Integration times were set to around 10 ms per pixel, and tapping amplitudes ranged between 70 and 80 nm.

## Supplementary Material



## Notes

Refractive index data of PMMA; membrane thickness considerations; numerical modeling for the magnetic qBIC; refractive index considerations; cross-sectional fabrication schematic; SEM image of a ripped, rectangular metasurface; AFM retrace curves; raw data for resonance scaling in Figure 4c; stability tests; simulated refractometric thin-film sensing (PDF)

## References

[1] Kildishev A. V., Boltasseva A., Shalaev V. M. (2013). Planar Photonics with Metasurfaces. Science.

[2] Yu N., Capasso F. (2014). Flat Optics with Designer Metasurfaces. Nat. Mater..

[3] Neshev D., Aharonovich I. (2018). Optical Metasurfaces: New Generation Building Blocks
for Multi-Functional Optics. Light Sci. Appl..

[4] Yu N., Genevet P., Kats M. A., Aieta F., Tetienne J.-P., Capasso F., Gaburro Z. (2011). Light Propagation
with Phase Discontinuities:
Generalized Laws of Reflection and Refraction. Science.

[5] Liu L., Zhang X., Kenney M., Su X., Xu N., Ouyang C., Shi Y., Han J., Zhang W., Zhang S. (2014). Broadband Metasurfaces
with Simultaneous Control of Phase and Amplitude. Adv. Mater..

[6] Arbabi A., Horie Y., Bagheri M., Faraon A. (2015). Dielectric Metasurfaces
for Complete Control of Phase and Polarization with Subwavelength
Spatial Resolution and High Transmission. Nat.
Nanotechnol..

[7] Balthasar
Mueller J. P., Rubin N. A., Devlin R. C., Groever B., Capasso F. (2017). Metasurface Polarization Optics: Independent Phase
Control of Arbitrary Orthogonal States of Polarization. Phys. Rev. Lett..

[8] Poulikakos L. V., Thureja P., Stollmann A., De Leo E., Norris D. J. (2018). Chiral
Light Design and Detection Inspired by Optical Antenna Theory. Nano Lett..

[9] Celebrano M., Wu X., Baselli M., Großmann S., Biagioni P., Locatelli A., De Angelis C., Cerullo G., Osellame R., Hecht B., Duò L., Ciccacci F., Finazzi M. (2015). Mode Matching in Multiresonant
Plasmonic Nanoantennas for Enhanced Second Harmonic Generation. Nat. Nanotechnol..

[10] Roger T., Vezzoli S., Bolduc E., Valente J., Heitz J. J. F., Jeffers J., Soci C., Leach J., Couteau C., Zheludev N. I., Faccio D. (2015). Coherent Perfect Absorption in Deeply
Subwavelength Films in the Single-Photon Regime. Nat. Commun..

[11] Chen W. T., Zhu A. Y., Capasso F. (2020). Flat Optics
with Dispersion-Engineered
Metasurfaces. Nat. Rev. Mater..

[12] Miroshnichenko A. E., Evlyukhin A. B., Yu Y. F., Bakker R. M., Chipouline A., Kuznetsov A. I., Luk’yanchuk B., Chichkov B. N., Kivshar Y. S. (2015). Nonradiating
Anapole Modes in Dielectric Nanoparticles. Nat.
Commun..

[13] Limonov M. F., Rybin M. V., Poddubny A. N., Kivshar Y. S. (2017). Fano Resonances
in Photonics. Nat. Photonics.

[14] Kravets V. G., Kabashin A. V., Barnes W. L., Grigorenko A. N. (2018). Plasmonic
Surface Lattice Resonances: A Review of Properties and Applications. Chem. Rev..

[15] Marinica D. C., Borisov A. G., Shabanov S. V. (2008). Bound States in
the Continuum in
Photonics. Phys. Rev. Lett..

[16] Hsu C. W., Zhen B., Stone A. D., Joannopoulos J. D., Soljačić M. (2016). Bound States in the
Continuum. Nat. Rev. Mater..

[17] Sadrieva Z., Frizyuk K., Petrov M., Kivshar Y., Bogdanov A. (2019). Multipolar
Origin of Bound States in the Continuum. Phys.
Rev. B.

[18] Liu Z., Xu Y., Lin Y., Xiang J., Feng T., Cao Q., Li J., Lan S., Liu J. (2019). High-Q Quasibound States in the Continuum
for Nonlinear Metasurfaces. Phys. Rev. Lett..

[19] Li Z., Kim M.-H., Wang C., Han Z., Shrestha S., Overvig A. C., Lu M., Stein A., Agarwal A. M., Lončar M., Yu N. (2017). Controlling Propagation
and Coupling
of Waveguide Modes Using Phase-Gradient Metasurfaces. Nat. Nanotechnol..

[20] Kodigala A., Lepetit T., Gu Q., Bahari B., Fainman Y., Kanté B. (2017). Lasing Action from Photonic Bound States in Continuum. Nature.

[21] Tittl A., Leitis A., Liu M., Yesilkoy F., Choi D.-Y., Neshev D. N., Kivshar Y. S., Altug H. (2018). Imaging-Based Molecular
Barcoding with Pixelated Dielectric Metasurfaces. Science.

[22] Romano S., Zito G., Torino S., Calafiore G., Penzo E., Coppola G., Cabrini S., Rendina I., Mocella V. (2018). Label-Free Sensing of Ultralow-Weight Molecules with
All-Dielectric Metasurfaces Supporting Bound States in the Continuum. Photonics Res..

[23] Yesilkoy F., Arvelo E. R., Jahani Y., Liu M., Tittl A., Cevher V., Kivshar Y., Altug H. (2019). Ultrasensitive Hyperspectral
Imaging and Biodetection Enabled by Dielectric Metasurfaces. Nat. Photonics.

[24] Jahani Y., Arvelo E. R., Yesilkoy F., Koshelev K., Cianciaruso C., De Palma M., Kivshar Y., Altug H. (2021). Imaging-Based
Spectrometer-Less
Optofluidic Biosensors Based on Dielectric Metasurfaces for Detecting
Extracellular Vesicles. Nat. Commun..

[25] Aigner A., Weber T., Wester A., Maier S. A., Tittl A. (2024). Continuous
Spectral and Coupling-Strength Encoding with Dual-Gradient Metasurfaces. Nat. Nanotechnol..

[26] Sortino L., Gale A., Kühner L., Li C., Biechteler J., Wendisch F. J., Kianinia M., Ren H., Toth M., Maier S. A., Aharonovich I., Tittl A. (2024). Optically Addressable
Spin Defects Coupled to Bound States in the Continuum Metasurfaces. Nat. Commun..

[27] Heimig, C. ; Antonov, A. A. ; Gryb, D. ; Possmayer, T. ; Weber, T. ; Hirler, M. ; Biechteler, J. ; Sortino, L. ; de S. Menezes, L. ; Maier, S. A. ; Gorkunov, M. V. ; Kivshar, Y. ; Tittl, A. Chiral Nonlinear Polaritonics with van Der Waals Metasurfaces. 2025, arXiv:2410.18760. arXiv. https://arxiv.org/abs/2410.18760. (accessed January 23, 2026). 10.1126/sciadv.aeb5631 PMC13025102 41894509

[28] Adi W., Rosas S., Beisenova A., Biswas S. K., Mei H., Czaplewski D. A., Yesilkoy F. (2024). Trapping Light in Air with Membrane
Metasurfaces for Vibrational Strong Coupling. Nat. Commun..

[29] Solntsev A. S., Agarwal G. S., Kivshar Y. S. (2021). Metasurfaces for Quantum Photonics. Nat. Photonics.

[30] Rodrigo D., Limaj O., Janner D., Etezadi D., García
De Abajo F. J., Pruneri V., Altug H. (2015). Mid-Infrared Plasmonic
Biosensing with Graphene. Science.

[31] Liang Y., Koshelev K., Zhang F., Lin H., Lin S., Wu J., Jia B., Kivshar Y. (2020). Bound States
in the Continuum in
Anisotropic Plasmonic Metasurfaces. Nano Lett..

[32] Aigner A., Tittl A., Wang J., Weber T., Kivshar Y., Maier S. A., Ren H. (2022). Plasmonic
Bound States in the Continuum
to Tailor Light-Matter Coupling. Sci. Adv..

[33] Koshelev K., Bogdanov A., Kivshar Y. (2019). Meta-Optics
and Bound States in the
Continuum. Sci. Bull..

[34] Li S., Zhou C., Liu T., Xiao S. (2019). Symmetry-Protected
Bound States in the Continuum Supported by All-Dielectric Metasurfaces. Phys. Rev. A.

[35] Kuznetsov A. I., Miroshnichenko A. E., Brongersma M. L., Kivshar Y. S., Luk’yanchuk B. (2016). Optically
Resonant Dielectric Nanostructures. Science.

[36] Baranov D. G., Zuev D. A., Lepeshov S. I., Kotov O. V., Krasnok A. E., Evlyukhin A. B., Chichkov B. N. (2017). All-Dielectric Nanophotonics: The
Quest for Better Materials and Fabrication Techniques. Optica.

[37] Sun S., Zhou Z., Zhang C., Gao Y., Duan Z., Xiao S., Song Q. (2017). All-Dielectric Full-Color Printing
with TiO2 Metasurfaces. ACS Nano.

[38] Kühner L., Sortino L., Tilmann B., Weber T., Watanabe K., Taniguchi T., Maier S. A., Tittl A. (2023). High-Q Nanophotonics
over the Full Visible Spectrum Enabled by Hexagonal Boron Nitride
Metasurfaces. Adv. Mater..

[39] Yang Y., Lee E., Park Y., Seong J., Kim H., Kang H., Kang D., Han D., Rho J. (2025). The Road to Commercializing
Optical Metasurfaces: Current Challenges and Future Directions. ACS Nano.

[40] Patoux A., Agez G., Girard C., Paillard V., Wiecha P. R., Lecestre A., Carcenac F., Larrieu G., Arbouet A. (2021). Challenges
in Nanofabrication for Efficient Optical Metasurfaces. Sci. Rep..

[41] Kühne J., Wang J., Weber T., Kühner L., Maier S. A., Tittl A. (2021). Fabrication Robustness
in BIC Metasurfaces. Nanophotonics.

[42] Xu G., Kang Q., Fan X., Yang G., Guo K., Guo Z. (2022). Influencing Effects
of Fabrication Errors on Performances of the
Dielectric Metalens. Micromachines.

[43] Green T. A. (2014). Gold Etching
for Microfabrication. Gold Bull..

[44] Pakpum C., Boonyawan D. (2020). Redeposition-Free
of Silicon Etching by CF4 Microwave
Plasma in a Medium Vacuum Process Regime. Surf.
Coat. Technol..

[45] Winder C. (2001). The Toxicology
of Chlorine. Environ. Res..

[46] Bertolini J. C. (1992). Hydrofluoric
Acid: A Review of Toxicity. J. Emerg. Med..

[47] Acikgoz C., Hempenius M. A., Huskens J., Vancso G. J. (2011). Polymers in Conventional
and Alternative Lithography for the Fabrication of Nanostructures. Eur. Polym. J..

[48] Chen Y. (2015). Nanofabrication
by Electron Beam Lithography and Its Applications: A Review. Microelectron. Eng..

[49] Su V.-C., Chu C. H., Sun G., Tsai D. P. (2018). Advances
in Optical
Metasurfaces: Fabrication and Applications. Opt. Express.

[50] Kim S. C., Lim B. O., Lee H. S., Shin D.-H., Kim S. K., Park H. C., Rhee J. K. (2004). Sub-100 Nm T-Gate Fabrication Using
a Positive Resist ZEP520/P­(MMA-MAA)/PMMA Trilayer by Double Exposure
at 50 kV e-Beam Lithography. Mater. Sci. Semicond.
Process..

[51] Gangnaik A. S., Georgiev Y. M., Holmes J. D. (2017). New Generation Electron Beam Resists:
A Review. Chem. Mater..

[52] Ao X., Wang D., Odom T. W. (2019). Enhanced
Fields in Mirror-Backed
Low-Index Dielectric Structures. ACS Photonics.

[53] Kim K.-H., Kim I.-P. (2022). Quasi-Bound States
in the Continuum with High Q-Factors
in Metasurfaces of Lower-Index Dielectrics Supported by Metallic Substrates. RSC Adv..

[54] Kulkarni S. P., Pathak A. K., Krishnaswamy S., Aydin K. (2025). High-Q Emission from
Colloidal Quantum Dots Embedded in Polymer Quasi-BIC Metasurfaces. Nano Lett..

[55] Kim M., Park N.-R., Yu A., Kim J. T., Jeon M., Jeon S.-W., Han S.-W., Kim M.-K. (2023). Multilayer All-Polymer
Metasurface Stacked on Optical Fiber via Sequential Micro-Punching
Process. Nanophotonics.

[56] Zhang L., Zhao Z., Tao L., Wang Y., Zhang C., Yang J., Jiang Y., Duan H., Zhao X., Chen S., Wang Z. (2024). A Review of
Cascaded Metasurfaces
for Advanced Integrated Devices. Micromachines.

[57] Martiradonna L., Carbone L., Tandaechanurat A., Kitamura M., Iwamoto S., Manna L., De Vittorio M., Cingolani R., Arakawa Y. (2008). Two-Dimensional Photonic Crystal
Resist Membrane Nanocavity
Embedding Colloidal Dot-in-a-Rod Nanocrystals. Nano Lett..

[58] Koshelev K., Lepeshov S., Liu M., Bogdanov A., Kivshar Y. (2018). Asymmetric
Metasurfaces with High-Q Resonances Governed by Bound States in the
Continuum. Phys. Rev. Lett..

[59] Ho Y.-L., Fong C. F., Wu Y.-J., Konishi K., Deng C.-Z., Fu J.-H., Kato Y. K., Tsukagoshi K., Tung V., Chen C.-W. (2024). Finite-Area Membrane
Metasurfaces
for Enhancing Light-Matter Coupling in Monolayer Transition Metal
Dichalcogenides. ACS Nano.

[60] Ma X., Kudtarkar K., Chen Y., Cunha P., Ma Y., Watanabe K., Taniguchi T., Qian X., Hipwell M. C., Wong Z. J., Lan S. (2022). Coherent Momentum Control of Forbidden
Excitons. Nat. Commun..

[61] Sakoda K. (1995). Symmetry,
Degeneracy, and Uncoupled Modes in Two-Dimensional Photonic Lattices. Phys. Rev. B.

[62] Zhen B., Hsu C. W., Lu L., Stone A. D., Soljačić M. (2014). Topological
Nature of Optical Bound States in the Continuum. Phys. Rev. Lett..

[63] Wood R. W. (1902). XLII. On
a Remarkable Case of Uneven Distribution of Light in a Diffraction
Grating Spectrum. London Edinb. Dublin Philos.
Mag. J. Sci..

[64] Strutt J. W. (1907). On the
Dynamical Theory of Gratings. Proc. R. Soc.
London Ser. Contain. Pap. Math. Phys. Character.

[65] Schmidt M., Boettger G., Eich M., Morgenroth W., Huebner U., Boucher R., Meyer H. G., Konjhodzic D., Bretinger H., Marlow F. (2004). Ultralow Refractive
Index Substrates–a
Base for Photonic Crystal Slab Waveguides. Appl.
Phys. Lett..

[66] Kim Y., Baek S., Gupta P., Kim C., Chang K., Ryu S.-P., Kang H., Kim W. S., Myoung J., Park W., Kim K. (2019). Air-like Plasmonics
with Ultralow-Refractive-Index
Silica Aerogels. Sci. Rep..

[67] Konishi K., Akai D., Mita Y., Ishida M., Yumoto J., Kuwata-Gonokami M. (2020). Circularly Polarized Vacuum Ultraviolet
Coherent Light
Generation Using a Square Lattice Photonic Crystal Nanomembrane. Optica.

[68] Kirchhoff G. (1850). Über
das Gleichgewicht und die Bewegung einer elastischen Scheibe. J. Für Reine Angew. Math. Crelles J..

[69] Timošenko, S. P. ; Woinowsky-Krieger, S. Theory of Plates and Shells, 2ed.; Engineering societies monographs; McGraw-Hill: New York, 1959; Vol. 2, p 20.

[70] Castellanos-Gomez A., Poot M., Steele G. A., van der Zant H. S. J., Agraït N., Rubio-Bollinger G. (2012). Elastic Properties
of Freely Suspended MoS2 Nanosheets. Adv. Mater..

[71] Jiang C., Markutsya S., Pikus Y., Tsukruk V. V. (2004). Freely Suspended
Nanocomposite Membranes as Highly Sensitive Sensors. Nat. Mater..

[72] Markutsya S., Jiang C., Pikus Y., Tsukruk V. V. (2005). Freely Suspended
Layer-by-Layer Nanomembranes: Testing Micromechanical Properties. Adv. Funct. Mater..

[73] Ciarella L., Tognazzi A., Mangini F., De Angelis C., Pattelli L., Frezza F. (2022). Finite-Size and Illumination Conditions
Effects in All-Dielectric Metasurfaces. Electronics.

[74] Sato R., Vinther Bertelsen C., Nikitin M., Lopez Aymerich E., Malureanu R., Edith Svendsen W., Lavrinenko A. V., Takayama O. (2024). Observation of Edge
Bound States in the Continuum at
Truncated Silicon Pillar Photonic Crystal. Nat.
Commun..

[75] Rahman F., Carbaugh D. J., Wright J. T., Rajan P., Pandya S. G., Kaya S. (2020). A Review of Polymethyl Methacrylate (PMMA) as a Versatile Lithographic
Resist – With Emphasis on UV Exposure. Microelectron. Eng..

[76] Liu W., Wang J., Xu X., Zhao C., Xu X., Weiss P. S. (2021). Single-Step Dual-Layer Photolithography for Tunable
and Scalable Nanopatterning. ACS Nano.

[77] Soci C., Hwang I.-W., Moses D., Zhu Z., Waller D., Gaudiana R., Brabec C. J., Heeger A. J. (2007). Photoconductivity
of a Low-Bandgap Conjugated Polymer. Adv. Funct.
Mater..

[78] Alshammari L. M., Almaymoni N. K., Shamsaldeen A. A., Mwafy E. A., Al-Ahmadi A. N., Abdelhameed D., Younes A. A. O., El-Tayeb Z., Alrajhi H., Nafee S. S., Mostafa A. M. (2026). Tailoring Dielectric and Optical
Properties of Polystyrene/PMMA Blends via Al2O3 Nanoparticles for
Optoelectronic and Microelectronic Applications. Radiat. Phys. Chem..

[79] Bückmann T., Stenger N., Kadic M., Kaschke J., Frölich A., Kennerknecht T., Eberl C., Thiel M., Wegener M. (2012). Tailored 3D
Mechanical Metamaterials Made by Dip-in Direct-Laser-Writing Optical
Lithography. Adv. Mater..

[80] Sortino L., Brooks M., Zotev P. G., Genco A., Cambiasso J., Mignuzzi S., Maier S. A., Burkard G., Sapienza R., Tartakovskii A. I. (2020). Dielectric
Nanoantennas for Strain Engineering in Atomically
Thin Two-Dimensional Semiconductors. ACS Photonics.

[81] Kim S., An S.-C., Kim Y., Shin Y. S., Antonov A. A., Seo I. C., Woo B. H., Lim Y., Gorkunov M. V., Kivshar Y. S., Kim J. Y., Jun Y. C. (2023). Chiral
Electroluminescence
from Thin-Film Perovskite Metacavities. Sci.
Adv..

[82] Fan S., Suh W., Joannopoulos J. D. (2003). Temporal
Coupled-Mode Theory for
the Fano Resonance in Optical Resonators. JOSA
A.

[83] Suh W., Wang Z., Fan S. (2004). Temporal Coupled-Mode
Theory and
the Presence of Non-Orthogonal Modes in Lossless Multimode Cavities. IEEE J. Quantum Electron..

