# Supplementary material for: Accessible, All-Polymer Metasurfaces: Low Effort, High Quality Factor
Source: ACS Nano. 2026 Feb 18;20(8):6722–31. doi: 10.1021/acsnano.5c15415 (PMC12961952; doi:10.1021/acsnano.5c15415)
Supplement: Supplementary file 1 [file nn5c15415_si_001.pdf]

Supporting Information for

Accessible, All-Polymer Metasurfaces: Low Effort, High Quality Factor

*Michael Hirler, Alexander A. Antonov, Enrico Baù, Andreas Aigner, Connor Heimig, Haiyang Hu and Andreas Tittl**

Chair in Hybrid Nanosystems, Nanoinstitute Munich, Faculty of Physics, Ludwig-Maximilians-Universität, Munich, Germany

* Email: Andreas.Tittl@physik.uni-muenchen.de.

Contents

Supplementary Note 1 – Refractive Index Data of PMMA 2
Supplementary Note 2 – Membrane Thickness Considerations 3
Supplementary Note 3 – Numerical Modelling for the Magnetic qBIC 4
Supplementary Note 4 – Refractive Index Considerations 5
Supplementary Note 5 – Cross-Sectional Fabrication Schematic 6
Supplementary Note 6 – SEM Image of Ripped, Rectangular Metasurface 7
Supplementary Note 7 – AFM Retrace Curves 8
Supplementary Note 8 – Raw Data for Resonance Scaling 9
Supplementary Note 9 – Stability Tests 10
Supplementary Note 10 – Simulated Refractometric Thin-Film Sensing 11

Supplementary Note 1 – Refractive Index Data of PMMA

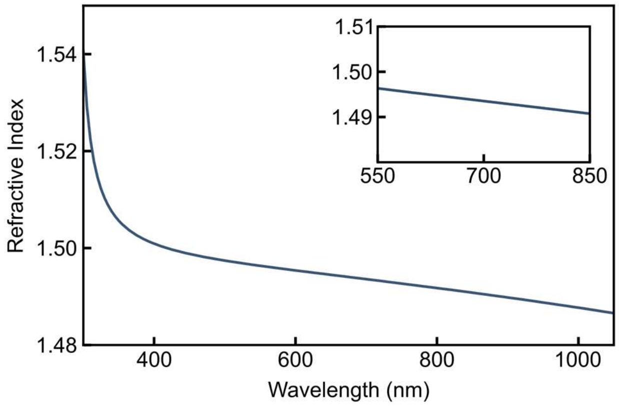

Figure S1. Real part of the refractive index of PMMA as determined in experiment. In the respective wavelength region, losses are negligible. A 300 nm thick PMMA film (AR-P 679-04, Allresist) was spin-coated on a silicon substrate (3600 rpm for 1 min) and measured by white-light spectral ellipsometry.

Supplementary Note 2 – Membrane Thickness Considerations

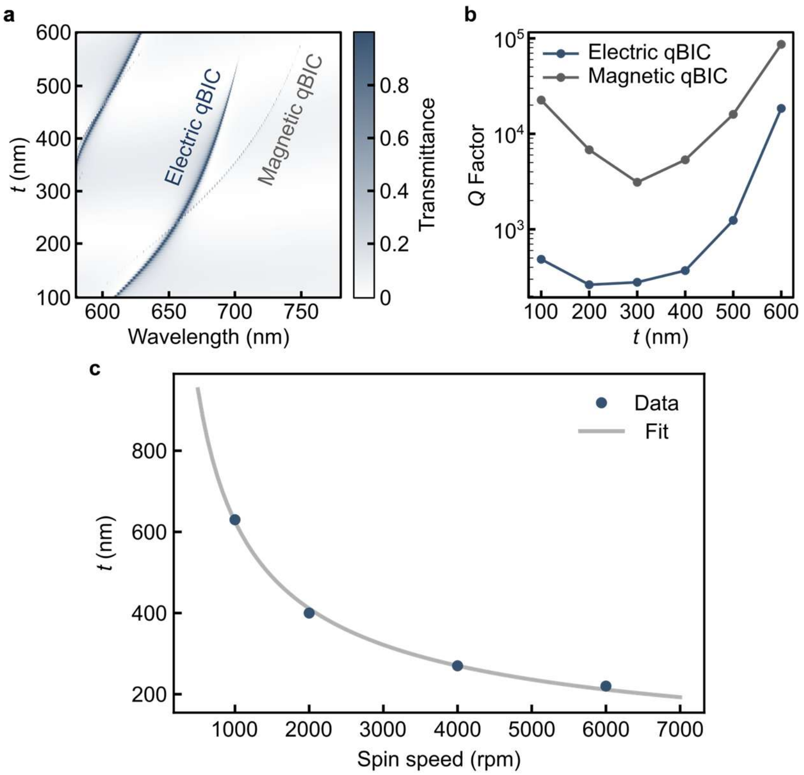

Figure S2. Membrane thickness considerations. (a) Simulated transmittance spectra depending on the thickness t of the membrane for $\alpha = 0.5$. (b) Corresponding Q factors for electric and magnetic qBICs. Since we assumed no intrinsic losses, they describe the radiative leakage channel (c) PMMA spin-curve obtained from fitting the data provided by the manufacturer[1] with equation $t = A\,\omega^{-p}$ (where ω denotes the spin-speed).

Supplementary Note 3 – Numerical Modelling for the Magnetic qBIC

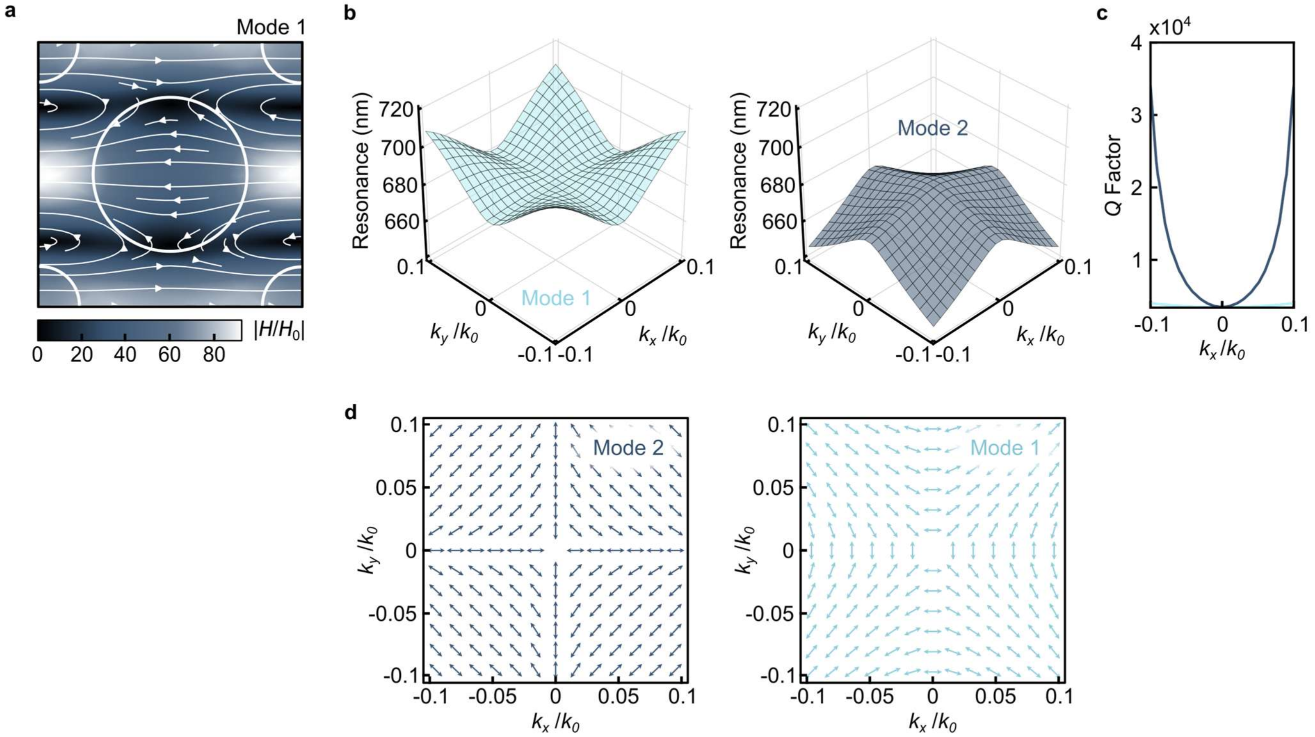

Figure S3. Numerical modelling for the magnetic BIC. (a) Magnetic near field enhancement $|H/H_0|$ as well as (b) resonance position and (c) Q factor in momentum space for the magnetic mode in Figure 2b. (d) Far field polarizations of the two magnetic modes with topological charges -1.

Supplementary Note 4 – Refractive Index Considerations

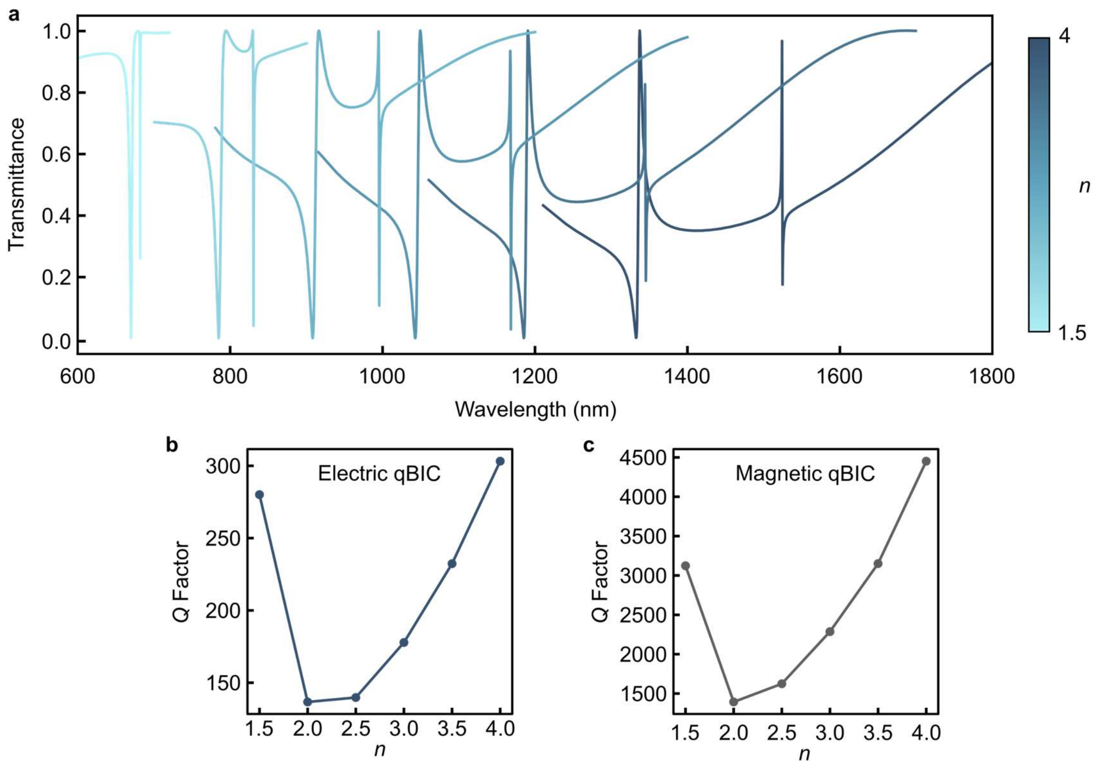

Figure S4. Refractive index considerations. (a) Simulated transmittance spectra for different values of the refractive index n of the resonator material for $\alpha = 0.5$. The corresponding radiative Q factors for the (b) electric and (c) magnetic qBICs.

Supplementary Note 5 – Cross-Sectional Fabrication Schematic

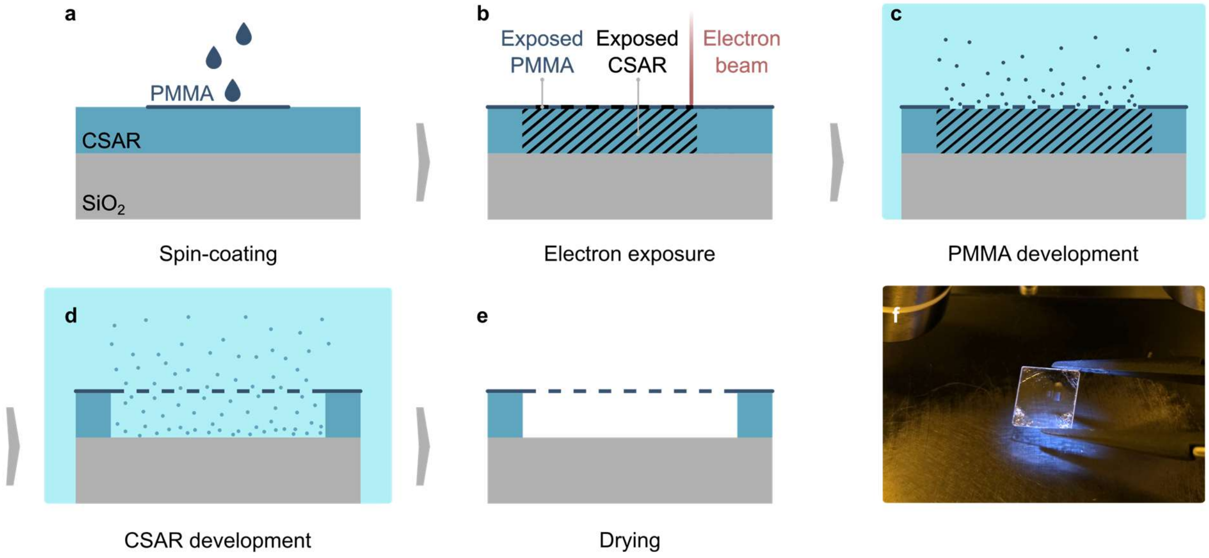

Figure S5. Cross-sectional fabrication schematic. (a) Successive spin-coating of polymer resists. (b) Single electron beam lithography step, simultaneously patterning PMMA while overexposing CSAR. (c) Selective development of PMMA. (d) Selective development of CSAR, removing the sacrificial layer through the porous PMMA and releasing the metasurface membrane. (e) Final structure after drying. (f) Photograph of the fabricated device. The metasurface pixels are visible due to their reflection.

Supplementary Note 6 – SEM Image of Ripped, Rectangular Metasurface

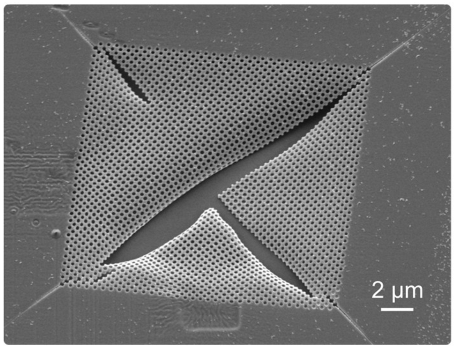

Figure S6. SEM image of ripped, rectangular metasurface. This demonstrates the tendency of the PMMA membranes to rip at the sharp corners. The viewing angle is 25°.

Supplementary Note 7 – AFM Retrace Curves

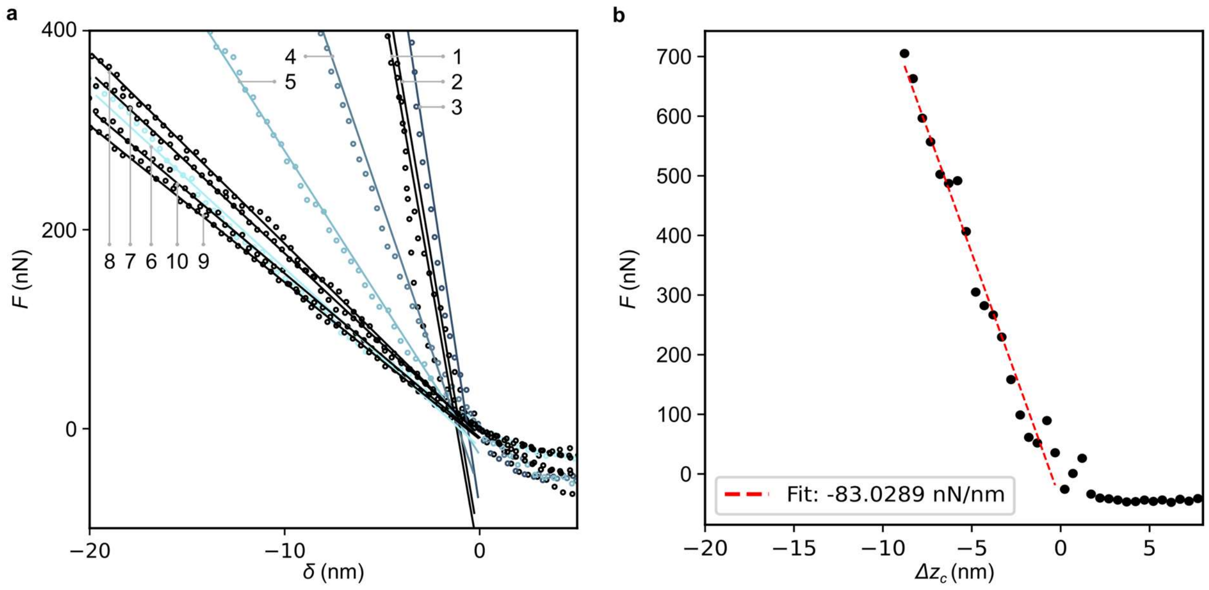

Figure S7. AFM retrace curves. (a) Force-displacement (F-δ) curves for all points in Figure 3e. The numbers indicate the position on the membrane as denoted in Figure 3c. (b) Force-displacement curve for silicon for determination of Δz_{c}. The lines denote fitting of eq (1).

Supplementary Note 8 – Raw Data for Resonance Scaling

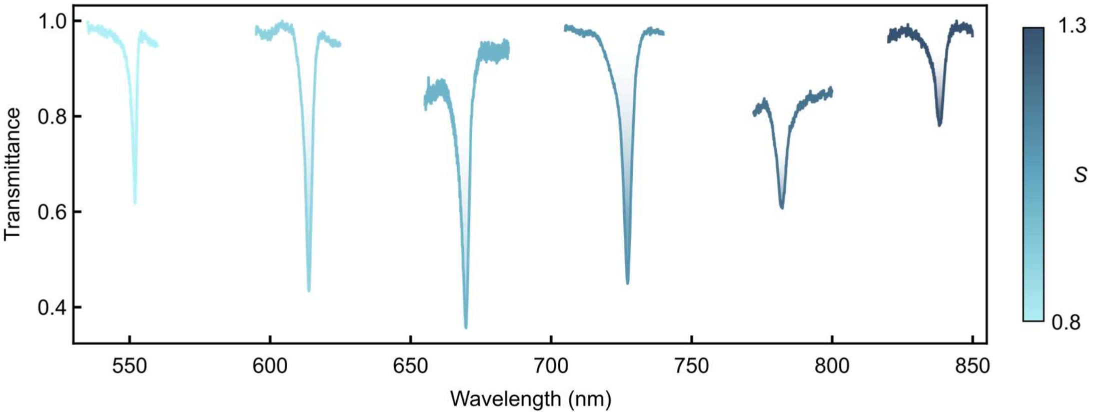

Figure S8. Raw transmittance spectra for resonance scaling without normalization. The spectra correspond to membranes with α = 0.5 and various lateral scaling factors S. In contrast, the spectra in Figure 4c in the main text were normalized such that the minimum and maximum transmittance equals 0 and 1, respectively.

Supplementary Note 9 – Stability Tests

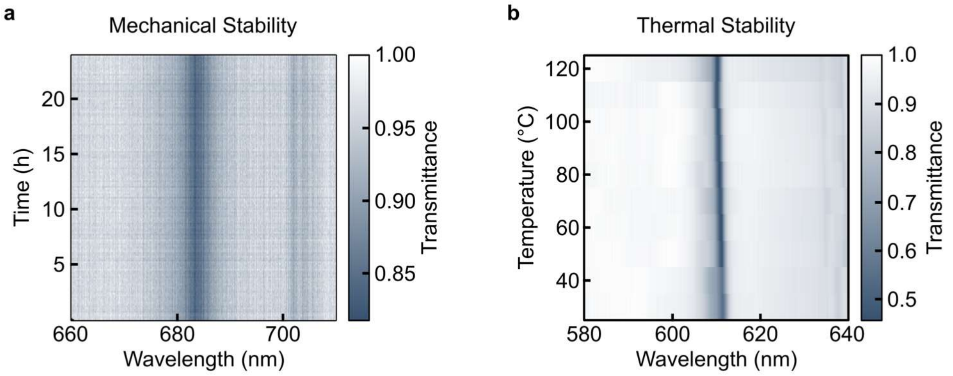

Figure S9. Stability tests. Experimental transmittance measurements for metasurfaces with scaling factors S as functions of (a) time for S = 1.0 and (b) temperature for S = 0.9.

Supplementary Note 10 – Simulated Refractometric Thin-Film Sensing

We numerically investigate the capabilities of the PMMA metasurface for label-free refractometric sensing of surface-adsorbed biomolecules such as proteins. To this end, we mimic a layer of biomolecules by coating the metasurface with a thin film of thickness t_{A} in the range of 1 to 10 nm and refractive index n_{A} = 1.4, congruent with previous findings.[2] In experiments, such a layer could be applied by drop casting different concentrations and subsequent evaporation of the solvent. Figure S10a shows a clear shift of the simulated qBIC wavelength upon analyte binding as a consequence of the change in RI of the environment. Since the RI contrast between analyte and resonator (RI of 1.5) is low, adding a thin film leads to similar effects as the scaling of the resonator geometry in Figure 4c. Figure S10b reveals a linear dependence between film thickness t_{A} and the resonance shift $\Delta\lambda$, which can be quantified via the surface sensitivity $S_{\mathrm{S}} = \frac{\Delta\lambda}{t_{\mathrm{A}}}$. We find S_{S} = 0.96 for α = 0.5 for the electric qBIC resonance.

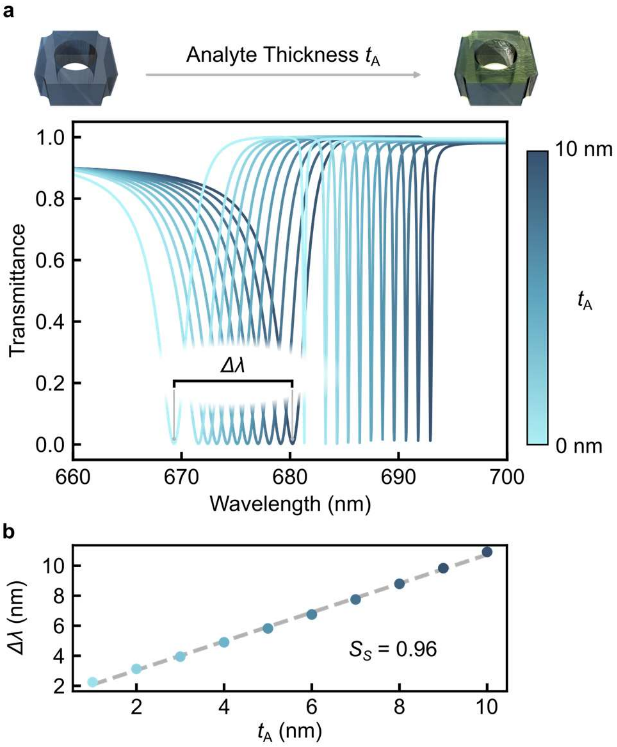

Figure S10. Simulated refractometric thin-film sensing. (a) The transmittance spectrum exhibits a red shift as the analyte thickness t_A increases. The analyte RI is fixed at n_A = 1.4. (b) The corresponding resonance shift $\Delta\lambda$ as a function of t_A. The dashed line indicates a linear fit whose slope yields a surface sensitivity of S_S = 0.96 for α = 0.5.

REFERENCES

(1) Allresist. AR-P 630-670 Produktinformation (Datasheet). Allresist GmbH **2014**, Datasheet, AR-P630/670.

(2) Cross, G. H.; Reeves, A. A.; Brand, S.; Popplewell, J. F.; Peel, L. L.; Swann, M. J.; Freeman, N. J. A New Quantitative Optical Biosensor for Protein Characterisation. *Biosens. Bioelectron.* **2003**, *19*, 383–390.
